# Enhanced Human Gingival Fibroblast Response and Reduced *Porphyromonas gingivalis* Adhesion with Titania Nanotubes

**DOI:** 10.1155/2020/5651780

**Published:** 2020-06-06

**Authors:** Zhiqiang Xu, Yuqi He, Xiufeng Zeng, Xiuxia Zeng, Junhui Huang, Xi Lin, Jiang Chen

**Affiliations:** ^1^Department of Stomatology, Affiliated Hospital of Putian University, Putian, Fujian 351100, China; ^2^Department of Oral Implantology, Affiliated Stomatological Hospital of Fujian Medical University, Fuzhou, Fujian 350002, China; ^3^Hanjiang Zeng Xiufeng Dental Clinic, Putian, Fujian 351117, China; ^4^Department of Stomatology, Putian Hanjiang Hospital, Putian, Fujian 351100, China; ^5^Laboratory of Electron Microscopy, Public Technology Service Center, Fujian Medical University, Fuzhou, Fujian 350002, China

## Abstract

Successful dental implants rely on stable osseointegration and soft-tissue integration. Titania nanotubes (TNTs) with a diameter of 100 nm could increase the mesenchymal stem cell response and simultaneously decrease *Staphylococcus aureus* adhesion. However, the interactions between the modified surface and surrounding soft tissues are still unknown. In the present study, we fully investigated the biological behavior of human gingival fibroblasts (HGFs) and the adhesion of *Porphyromonas gingivalis* (*P. gingivalis*). TNTs were synthesized on titanium (Ti) surfaces by electrochemical anodization at 10, 30, and 60 V, and the products were denoted as NT10, NT30, and NT60, respectively. NT10 (diameter: 30 nm) and NT30 (diameter: 100 nm) could enhance the HGF functions, such as cell attachment and proliferation and extracellular matrix- (ECM-) related gene expressions, with the latter showing higher enhancement. NT60 (diameter: 200 nm) clearly impaired cell adhesion and proliferation and ECM-related gene expressions. Bacterial adhesion on the TNTs decreased and reached the lowest value on NT30. Therefore, NT30 without pharmaceuticals can be used to substantially enhance the HGF response and reduce *P. gingivalis* adhesion to the utmost, thus demonstrating significant potential in the transgingival part of dental implants.

## 1. Introduction

Titanium (Ti) implants are widely used in dentistry to replace missing teeth due to their excellent material properties and biological activity [1, 2]. However, given the exposure of the transgingival part to the oral environment, Ti implants are easily invaded by bacteria at implant-soft tissue sites. Bioactive molecules and antibacterial agents are introduced into the transgingival part to improve the soft-tissue integration and antibacterial activity [3, 4]. However, these coatings are expensive and difficult to fabricate and also unsuitable for conventional implants with cylindrical or tapered shapes [5, 6]. Therefore, the application of the transgingival part of dental implants with excellent soft-tissue integration and antibacterial property without pharmaceuticals is desirable.

Given the nanoscale dimensions of the physiological extracellular matrix (ECM), titania nanotubes (TNTs) manufactured on the Ti surface for the transgingival part of dental implants have received considerable attention [4, 7]. As the major cells in peri-implant soft tissue, human gingival fibroblasts (HGFs) can secrete ECM containing abundant collagen fibers, which contribute to gingival wound healing repair and regeneration [4]. Therefore, the investigation of the biological response of HGFs toward TNTs while assessing the interactions between TNTs and soft tissues was proposed. TNTs with a diameter of 70-90 nm have been reported to promote the cell adhesion and proliferation of HGFs and ECM-related gene expressions [3, 8]. However, TNTs with a diameter of 50-100 nm have also been demonstrated to support the adhesion and proliferation of HGFs but without notable promotion [9, 10]. TNTs with diameters of 15-100 nm even seriously inhibited the adhesion and spread of HGFs, and the inhibitory effect increased with the increased diameter of TNTs [11]. In addition, 100 nm should not be the diameter threshold of TNTs to exhibit effects on the cell behavior. However, TNTs with a diameter of >100 nm are difficult to prepare using traditional strong acid electrolytes.

Bacteria invade the implant surface during surgical operation and wound healing; thus, “the race for the surface” starts before soft-tissue integration occurs [12]. Thus, the potential antibacterial properties of dental implants are critical given the abundance of pathogens in the oral environment and inherent nonantibacterial nature of Ti. We have previously shown that TNTs could decrease the adhesion of Gram-positive *Staphylococcus aureus* even without drugs, which could present harmful side effects [13]. However, whether the result can be applied to other kinds of bacteria, such as the Gram-negative bacterium *Porphyromonas gingivalis* (*P. gingivalis*, one of the most common bacteria in peri-implantitis), is still unknown. TNTs alone also been demonstrated to increase both the adhesion of Gram-positive and that of Gram-negative bacteria, and this action might be due to the differences in their physical and chemical features [3]. Thus, the same TNTs for both HGFs and *P. gingivalis* should be assessed given the conflicting antibacterial assays.

Here, we aimed to manufacture optimum TNTs that enhance HGF response and simultaneously reduce *P. gingivalis* adhesion without pharmacological therapy. For this purpose, TNTs with three different diameters were manufactured using a weak acidic electrolyte, which can generate smoother and larger nanotubular layers within a suitable range of voltage compared with a strong acid electrolyte. For the first time, the response of HGFs and the adhesion of *P. gingivalis* on the three TNTs were systematically and simultaneously studied.

## 2. Materials and Methods

### 2.1. Preparation of TNTs on Ti

TNTs were prepared via anodization on a Ti sheet. A 1 × 1 cm^2^ Ti sheet was polished with a gradient of sandpapers in the same direction to create a grooved microrough surface and degreased by sonication in acetone and deionized water for 10 min. The sheet was then eroded in 4 wt% HF-5 mol/L HNO_3_, rinsed with distilled water, and dried in air. As previously described, anodization was conducted using a mixture of 0.50 wt% NH_4_F+10 vol% H_2_O in glycerol at 10, 30, and 60 V (the samples were denoted as NT10, NT30, and NT60, respectively) for 3 h and ultimately annealed in air for 2 h at 450°C [13]. An identically sized Ti sheet with a native TiO_2_ oxide layer was used as the control. All samples were sterilized using ultraviolet irradiation (0.5 h for either side) before use.

### 2.2. Specimen Characterization

#### 2.2.1. Scanning Electron Microscopy (SEM)

The surface morphology of the samples was observed by SEM. All samples were sputter-coated with platinum before observation.

#### 2.2.2. Phase Analysis

The crystalline phase analysis of the samples was detected using X-ray diffraction (XRD).

#### 2.2.3. Atomic Force Microscopy (AFM)

Surface feature and roughness were examined with AFM in air tapping mode, with a scan size of 100 *μ*m.

#### 2.2.4. Contact Angle Determination

A 1 *μ*L sessile droplet of double distilled water was delivered to the specimen surface. Contact angles were measured using a drop shape analysis system.

#### 2.2.5. Total Proteins and Fibronectin (FN) Adsorption Assay

A 1 mL droplet of Dulbecco's modified Eagle's medium-low glucose containing 10% fetal bovine serum (FBS) was pipetted onto each specimen in 24-well plates. All specimens were transferred to new 24-well plates and gently rinsed with phosphate-buffered saline (PBS) after 2 h. The total proteins adsorbed onto the samples were determined using a Micro BCA protein assay kit. The FN adsorbed onto the samples was determined using an enzyme-linked immunosorbent assay kit for FN.

### 2.3. Cell Cultures

HGFs were obtained from the Key Laboratory of Stomatology, Fujian Medical University. As previously described, the cells were obtained from healthy gingival tissues of orthodontic patients who had their premolar teeth removed [14]. Tissues from different donors were minced to approximately 1 × 1 mm^2^ and then maintained in Dulbecco's modified Eagle's medium-low glucose supplemented with 10% FBS and 1% penicillin streptomycin. HGFs were digested and subcultured at a 1 : 3 ratio after reaching 80% confluence. Cells between passage 2 and passage 5 were used for the experiments. HGFs were seeded onto each sample in 24-well plates at a density of 4 × 10^4^/well for the cell adhesion assay and 2 × 10^4^/well for the other assays.

#### 2.3.1. Cell Morphology

After culturing for 2 days, all the samples were fixed with 2.5% glutaraldehyde overnight. The samples were dehydrated in graded ethanol series and freeze-dried. All samples were sputter-coated with platinum before SEM observation.

#### 2.3.2. Adhesion and Proliferation

For the cell adhesion assay, the cell nuclei were stained with 4,6-diamidino-2-phenylindole (DAPI) after 2 h of incubation and then observed under a fluorescence microscope. For the cell proliferation assay, the cells were evaluated using Cell Counting Kit-8 (CCK-8) assay after 1, 3, and 7 days of incubation.

#### 2.3.3. Gene Expressions

The relative gene expression levels of HGFs, including integrin *β*1 (ITGB1, an important receptor mediating the cellular binding onto the implant surface) [15], FN (an important adhesion protein) [16], vinculin (VCL, an important cytoskeleton binding protein associated with adhesion strength and cell migration) [17], and type I collagen (COL1, the main component in gingival tissues) [18, 19], were measured using quantitative reverse transcription polymerase chain reaction (qRT-PCR). The total RNA was isolated from the cells by using Trizol reagent after being cultured for 7 days, and the cDNA was synthesized from the total RNA by using a cDNA Reverse Transcription Kit in accordance with the manufacturer's instructions. The qRT-PCR analysis was conducted on an ABI Prism 7500 real-time PCR cycler with SYBR Premix Ex Taq™ II.  Table 1 shows the primer sequences of the genes. The mRNA levels for cells were normalized for glyceraldehyde-3-phosphate dehydrogenase (GAPDH) mRNA.

### 2.4. Bacterial Culture

*P. gingivalis* (ATCC33277) was cultivated in brain heart infusion broth with 5% yeast extract, 1% hemin, and 0.2% vitamin K1 at 37°C under standard anaerobic conditions. The TNTs and control Ti were all separately placed in 24-well plates and incubated in 1 mL bacteria-containing medium (10^6^ CFU/mL).

#### 2.4.1. Bacterial Morphology

All samples were fixed with 2.5% glutaraldehyde for 4 h, dehydrated in graded ethanol series, and sputter-coated with platinum for SEM observation.

#### 2.4.2. Antibacterial Assay

In vitro antibacterial activity was assessed by a plate-counting method after culturing for 6 h. The sample was ultrasonically agitated to detach the bacteria from the sample, and the viable bacteria in PBS were recultivated on agar plates for colony counting through 3 days of anaerobic incubation at 37°C.

### 2.5. Statistical Analysis

All the data were expressed as the means ± standard deviation (*N* = 3) for each experiment. The level of significance was determined by the analysis of variance, followed by Student–Newman–Keuls post hoc test for multiple comparisons. *p* < 0.05 was considered significant, and *p* < 0.01 was considered highly significant. Significance levels were ^∗^*p* < 0.05 and ^∗∗^*p* < 0.01 when compared with Ti, ^#^*p* < 0.05 and ^##^*p* < 0.01 when compared with NT10, and ^&^*p* < 0.05 and ^&&^*p* < 0.01 when compared with NT30.

## 3. Results

### 3.1. Characterization of Specimens

#### 3.1.1. SEM

**Figure 1:**
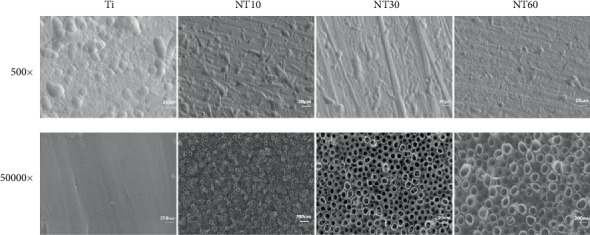
SEM images of different samples with two different magnifications of 500x and 50,000x [13].

As shown by the SEM images in Figure 1, pure Ti possessed random microrough surface features at low magnification and lacked evident nanotubes at high magnification. By contrast, NT10, NT30, and NT60 showed groove-like topography at low magnification and highly ordered nanotubes with diameters of approximately 30, 100, and 200 nm, respectively, at high magnification, just as shown in our previous work [13], thereby resulting in a dual micro- and nanorough surface.

#### 3.1.2. XRD

**Figure 2:**
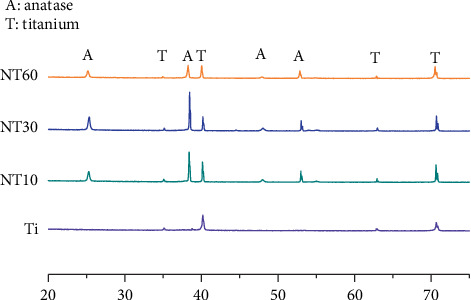
XRD patterns of TNTs after heat treatment at 450°C for 2 h and control Ti before heat treatment.

XRD patterns (Figure 2) showed that all three TNTs featured Ti and anatase peaks without any rutile nor amorphous peaks after annealing in air at 450°C for 2 h. Compared with the rutile and amorphous crystal types, the anatase crystal type possessed good antibacterial property and may improve the biocompatibility by favoring protein adhesion [20, 21].

#### 3.1.3. AFM

**Figure 3:**
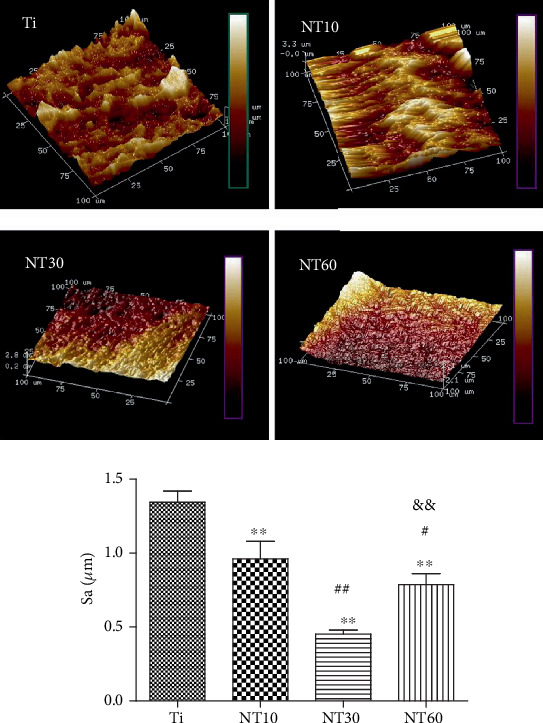
AFM images of different samples and multiple comparison results of surface roughness (Sa) on different samples.

The topographical features were evaluated by AFM (Figure 3). The prepared TNTs possessed a mechanically prepared groove-like topography, whereas the pure Ti featured a random micron-size rough topography. The roughness values of the samples decreased in the following order: Ti > NT10 > NT60 > NT30.

#### 3.1.4. Static Contact Angles

**Figure 4:**
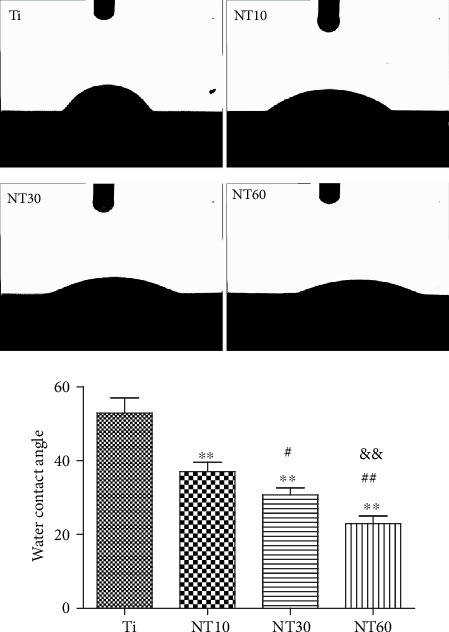
Images of water droplets on different samples and multiple comparison results of contact angles on different samples.

The wettability of samples was detected by measuring the water contact angles (Figure 4). The TNTs showed favorable hydrophilia with a relatively low water contact angle, and the hydrophilicity increased with the increase in diameter. By contrast, pure Ti was hydrophobic.

#### 3.1.5. Total Proteins and FN Adsorption Assay

**Figure 5:**
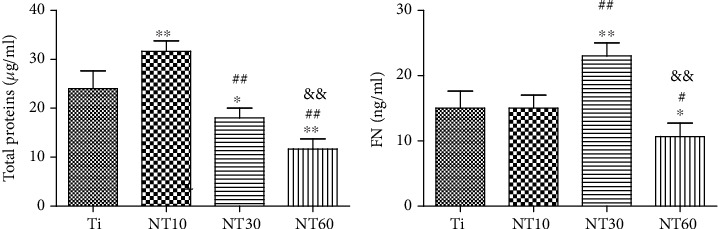
Absorbed total proteins and FN after 2 h of immersion in medium containing 10% FBS.

Figure 5 shows the amounts of adsorbed total proteins and FN from 10% FBS after 2 h of immersion. Compared with Ti, NT10 absorbed more total proteins whereas NT30 and NT60 absorbed less amount of proteins. NT30 absorbed more FN than Ti and NT10. Meanwhile, NT60 absorbed the least amount of FN.

### 3.2. In Vitro Biocompatibility Studies

#### 3.2.1. Cell Morphology

**Figure 6:**
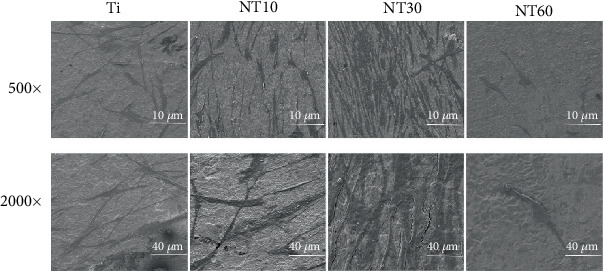
SEM images with two different magnifications of 500x and 2,000x showing the morphologies of HGFs after 2 days of culture on specimens.

The HGFs cultured on different surfaces exhibited noticeably varying shapes (Figure 6). Most of the HGFs on control Ti exhibited an oval shape without the noticeable filopodia extensions and had an irregular arrangement. The cells on NT10 and NT30 elongated further and featured a large number of prominent filopodia and lamellipodia extensions and were arranged orderly along the direction of the grooves, especially on NT30. The cells on NT60 were sparsely distributed and retained their slender shape without noticeable filopodia extensions.

#### 3.2.2. Adhesion and Proliferation

**Figure 7:**
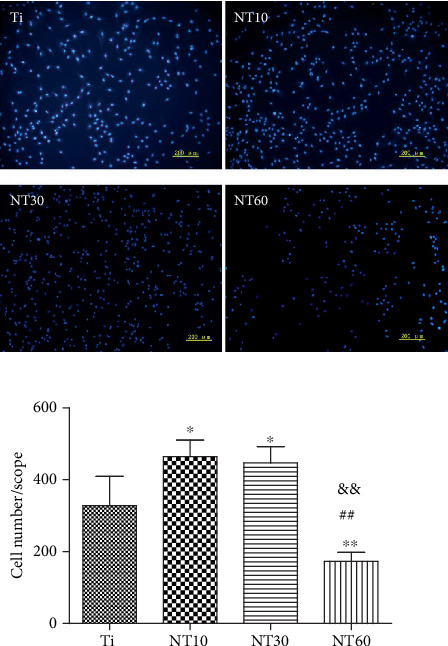
Fluorescence images of initial adherent HGFs stained with DAPI and cell numbers measured by counting cells after 2 h.

**Figure 8:**
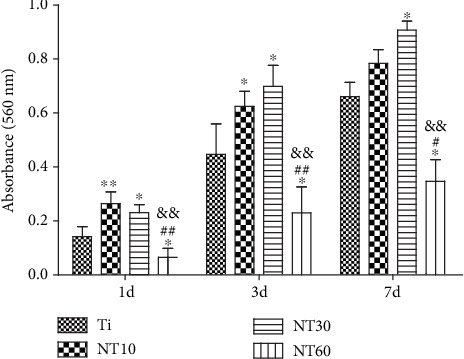
Cell proliferation measured by CCK-8 assay after culturing HGFs on different samples for 1, 3, and 7 days.

The initial adherent cell number was surveyed using DAPI staining (Figure 7). Cell attachment was significantly improved on NT10 and NT30 but was severely inhibited on NT60. Cell proliferation was surveyed using the CCK-8 assay (Figure 8). Cell proliferation on all surfaces increased with time. NT10 and NT30 could promote cell proliferation when compared with control Ti, with the latter showing a stronger promotion, although no statistically significant difference was found between Ti and NT10 on day 7. By contrast, cell attachment was severely inhibited on NT60.

#### 3.2.3. Gene Expressions

**Figure 9:**
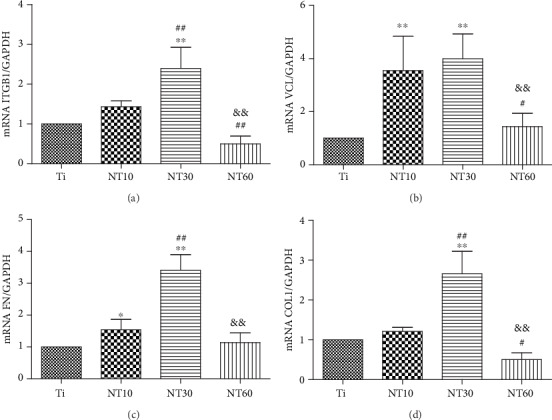
Relative expressions of (a) ITGB1, (b) VCL, (c) FN, and (d) COL1 by HGFs seeding on different substrates for 7 days.

The expression levels of ECM-related genes, including those of ITGB1, VCL, FN, and COL1, were assessed by qRT-PCR (Figure 9). The expression levels of relevant genes became increasingly higher with the increased diameter of NT10 and NT30, although no significant difference in the expressions of ITGB and COL1 was observed between NT10 and control Ti. However, the expression levels of ECM-related genes were downregulated on NT60.

### 3.3. Antibacterial Activity

**Figure 10:**
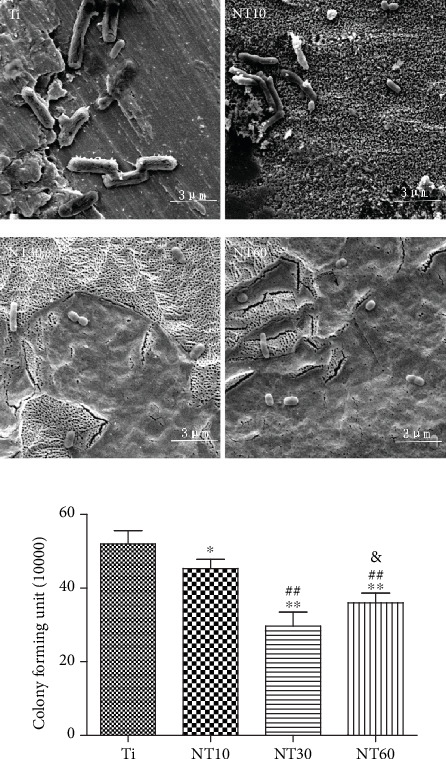
SEM images and quantitative analysis of viable adherent *P. gingivalis* on the samples after 6 h.

As shown in Figure 10, *P. gingivalis* displayed their normal rod shapes on control Ti and partly shrunk to round shapes and were distributed sparsely on TNTs, especially on NT30 and NT60. The number of viable bacteria on the samples decreased in the following order: Ti > NT10 > NT60 > NT30.

## 4. Discussions

The successful long-term function of a dental implant relies not only on the ideal osseointegration but also on the effective interaction of the implant with the surrounding soft tissues [22]. Soft-tissue integration with the dental implant establishes a biological seal between the oral environment and the implant. This characteristic inhibits unfavorable soft tissue recession and marginal bone resorption and contributes to the resistance against bacterial invasion [17]. However, although many studies have focused on the osseointegration of the body surface, few studies have been conducted for the design of transgingival surface. Many studies have recently shown that a microrough surface could establish a more stable soft-tissue attachment compared with the traditionally smooth surface and is thus more suitable for the transgingival part of dental implant [23]. In this work, TNTs with three different diameters were manufactured on the microrough Ti sheet, and the microrough Ti sheet with a native TiO_2_ oxide layer was used as the control. We previously confirmed that TNTs with a diameter of 100 nm could increase the response of mesenchymal stem cells and simultaneously decrease the adhesion of *S. aureus* [13]. Thus, in this study, we fully investigated the biological behavior of HGFs and the adhesion of *P. gingivalis*, which determined the peri-implant soft-tissue integration.

Upon contact of an implant surface with blood, plasma proteins are spontaneously adsorbed onto the implant surface within seconds and convey the effect of topographical cues to the attached cells/tissues to direct the biological performance of biomaterials [24, 25]. NT10 (diameter: 30 nm) with higher hydrophilicity induced more total proteins and thus improved the cell attachment at 2 h compared with Ti. The total protein aggregates (≈30 nm-size regime) are too small to anchor on NT30 (diameter: 100 nm) and NT60 (diameter: 200 nm), although both are more hydrophilic than Ti [13, 25]. However, plasma FN (≈120 nm-size regime) [26, 27], which plays an important role in the adhesion of HGFs onto the implant surface, could be more easily adsorbed onto NT30 than onto the other samples (Figure 4). Thus, NT30 induced more cell attachments than the other samples possibly because of the increased FN adhesions. However, NT60 induced less amounts of total proteins and FN and thus less achieved cell attachment than Ti. Furthermore, the TNTs showed lower roughness values than Ti, with NT30 achieving the lowest value. Fibroblasts are sensitive not only to the changes in hydrophilicity but also to those in Sa which influences the assembly of focal adhesion (FA) and subsequent intracellular signaling cascade activation [28, 29]. Only the roughness of NT30 was less than 0.5 *μ*m (Figure 3), whereas the surface with a roughness less than 0.5 *μ*m was considered to feature a smooth surface [30]. This finding is consistent with the conclusion that highly ordered and smooth TNTs could be fabricated by applying a suitable range of voltage, and the TNTs would become extremely compact or spongy-like and porous if the applied potential voltage was notably low or extremely high [31]. Given its low roughness, NT30 in this study might benefit the attachment of HGFs, because HGFs tend to adhere to smooth surfaces [32, 33]. The cell proliferation assay results showed that NT10 and NT30 could promote the cell proliferation compared with control Ti, with NT30 showing the stronger promotion. This finding is due to not only additional cell adhesions but also the increased surface area available for cell colonization and increased fluid exchange. The proliferation on NT60 was significantly inhibited, because extremely low protein amounts and cell adhesion could potentially lead to cell quiescence and apoptosis (oval-shaped HGFs without noticeable extensions on NT60 as shown in Figure 6, indicating cell death or cells with low activity).

HGFs cultured on NT10 and NT30 extended across the tubes to reach a protein-deposited surface for initial contact, thus further elongating and showing a large number of prominent filopodia and lamellipodia extensions according to the SEM images (Figure 6). An extensively stretched appearance indicated evidence of migration and activation [34]. The cells were also aligned in the same direction, which could be related to the underlying micro-/nanorough groove-like topographical features [35, 36]. The anisotropic groove-like surface texture created mechanical stress, which directed the cell fate via contact guidance, i.e., the attachment, proliferation, spread, migration, and alignment of cells [37]. Cell alignment plays a critical role in various cell behaviors, including membrane protein relocation, cytoskeleton reorganization, related gene expression, and ECM remodeling [14, 38]. It has been demonstrated that HGFs attach to the material surface through FAs, which anchor the cells to the substratum and can mediate both mechanical and biochemical signaling [39]. Thus, we investigated the expression levels of ECM-related genes, including those of ITGB, VCL, FN, and COL1. Our results showed that NT10 and NT30 significantly promoted the expressions of ECM-related genes and demonstrated excellent activity, with the latter exhibiting a higher promotion. HGFs on NT60 retained their oval shape without noticeable filopodia extension, indicating cell death or cells with a low activity, and thus the lack of noticeable related gene expressions. This finding is in accordance with the discovery that fibroblasts respond to surface topography by changes in the alignment and subsequent ECM production [9, 35]. FN, which occurs in two forms, namely, the soluble plasma FN in blood and insoluble cellular FN in ECM or on cell surfaces, is a key factor in the compatibility of dental implant materials for connective tissues [40]. VCL is a FA linker protein, and increased VCL expression is associated with enhanced adhesion strength [41]. The high expressions of cellular FN and VCL might also facilitate the adhesion of HGFs on NT10 and NT30. Furthermore, the groove-like surface texture on NT10 and NT30 could also contribute to promote more adhesion and proliferation via contact guidance.

The early failure of dental implants mostly results from bacteria and plaque formation on the implant surfaces before soft-tissue integration occurs, especially in the cases of periodontal disease [42]. Given the difficulty of killing bacteria after binding to the implant surface, prevention strategies have therefore become extremely important [43]. In our previous study, TNTs exhibited higher hydrophilicity and bioactivity in the anatase-phase after heat treatment, thereby decreasing the adhesion of *S. aureus* [13]. In this work, TNTs demonstrated the same characteristics by contact angle determination and phase analysis, thus reducing bacterial attachment. The bacteria shrunk to round shapes and were distributed sparsely, indicating their bacteria inactivation. This finding is consistent with our previous work. NT30 achieved the highest reduction of bacterial adhesion in this work, whereas in our previous research, this finding was observed with NT60. This finding is probably due to the use of Gram-negative *P. gingivalis* in the present work, whereas the Gram-positive *S. aureus* was used in our previous research. Surface properties, including roughness, hydrophilicity, and chemical composition, all affect the adhesion of bacteria [44]. However, the initial bacterial adhesion, especially that of Gram-negative bacteria with a thinner cell wall, is more easily influenced by roughness than by hydrophilicity [45]. Thus, NT30 caused the most reduction in the adhesion of *P. gingivalis* possibly because of its roughness (Sa < 0.5 *μ*m), which was the lowest detected by AFM. The obtained results are consistent with those obtained by a previous study, in which the smooth surface of machined neck (Sa < 0.5 *μ*m) was generally conducive for the attachment of soft tissue while contributing to the inhibition of bacterial biofilm adhesion [46].

## 5. Conclusion

The amount and bioactivity of HGFs existing in the implant-mucosa interface are important in the formation of soft-tissue seals, which could block bacteria from entering the space between the transgingival part and the biological tissue [17, 47]. NT30 could bioactivate HGF functions to the utmost, including not only cell attachment and proliferation but also ECM-related gene expressions. Bioactivating HGFs could increase the possibility to win the “race for the surface” against bacteria at the attachment sites. Moreover, NT30 could also reduce the adhesion of *P.* gingivalis to the transgingival part, facilitating the attachment of soft tissue, inhibiting the adhesion of bacterial biofilm, and preserving the crestal bone. Findings from previous and current studies indicate that NT30 might feature considerable potential in the intraosseous and transgingival parts of dental implants. Further studies are needed to investigate the in vivo and clinical implications when the results are applied for clinical use.

## Figures and Tables

**Table 1:** Primers used for qRT-PCR.

Gene	Forward primer sequence (5′-3′)	Reverse primer sequence (5′-3′)
ITGB1	GAATGCCTACTTCTGCACGAT	TTCCTTTGCTACGGTTGGTTA
VCL	CTCGTCCGGGTTGGAAAAGAG	AGTAAGGGTCTGACTGAAGCAT
FN	AGGAAGCCGAGGTTTTAACTG	AGGACGCTCATAAGTGTCACC
COLI	CGAAGACATCCCACCAATCA	GATCACGTCATCGCACAACA
GAPDH	TGGGTGTGAACCATGAGAAGT	TGAGTCCTTCCACGATACCAA

## Data Availability

The data used to support the finding of this study are included within the article.
